# Antimicrobials and Food-Related Stresses as Selective Factors for Antibiotic Resistance along the Farm to Fork Continuum

**DOI:** 10.3390/antibiotics10060671

**Published:** 2021-06-04

**Authors:** Federica Giacometti, Hesamaddin Shirzad-Aski, Susana Ferreira

**Affiliations:** 1 Department of Veterinary Medical Sciences, University of Bologna, Ozzano Emilia, 40064 Bologna, Italy; federica.giacometti3@unibo.it; 2 Infectious Diseases Research Center, Golestan University of Medical Sciences, Gorgan 49178-67439, Iran; shirzad_hessam@yahoo.com; 3 CICS-UBI-Centro de Investigação em Ciências da Saúde, Universidade da Beira Interior, 6200-506 Covilhã, Portugal

**Keywords:** antimicrobial resistance, food chain, stressors, cross-resistance, adaptive response

## Abstract

Antimicrobial resistance (AMR) is a global problem and there has been growing concern associated with its widespread along the animal–human–environment interface. The farm-to-fork continuum was highlighted as a possible reservoir of AMR, and a hotspot for the emergence and spread of AMR. However, the extent of the role of non-antibiotic antimicrobials and other food-related stresses as selective factors is still in need of clarification. This review addresses the use of non-antibiotic stressors, such as antimicrobials, food-processing treatments, or even novel approaches to ensure food safety, as potential drivers for resistance to clinically relevant antibiotics. The co-selection and cross-adaptation events are covered, which may induce a decreased susceptibility of foodborne bacteria to antibiotics. Although the available studies address the complexity involved in these phenomena, further studies are needed to help better understand the real risk of using food-chain-related stressors, and possibly to allow the establishment of early warnings of potential resistance mechanisms.

## 1. Introduction

In recent years, there has been an effort to reduce foodborne diseases, by the implementation of food safety measures from farm to fork. Nonetheless, a high burden of foodborne diseases still exists, with the World Health Organization estimating that each year worldwide, the consumption of unsafe food causes about 600 million cases of foodborne diseases and 420,000 deaths [1]. Further, amongst the cases of foodborne diseases, the ones caused by antibiotic-resistant bacteria are increasing and are a major health problem, with the food chain being pointed to as a relevant vehicle of antibiotic resistance to humans [2].

The use and misuse of antimicrobial compounds have been related to an increase in the emergence of antimicrobial resistance (AMR) amongst foodborne microorganisms. The identification of resistant microorganisms at every stage of the food chain, from farm to fork, highlights the major concern that is AMR [2,3]. The cross-adaptation and selective pressure exercised by antibiotics and biocides are considered as a key motive power for the emergence and spread of antibiotic resistance along the food chain [4]. However, other factors, such as different antimicrobial approaches, or even agricultural or food-processing procedures, may have a role in the emergence and spread of antibiotic resistance along the food chain.

During food production and processing, different types of antimicrobials are used throughout the several stages of the food chain, namely, antibiotics, agricides and biocides, among others (e.g., agrochemicals, feed and food preservatives, decontaminants, or disinfectants). These products are applied to ensure food quality and safety, as well as to assure the efficiency of the system. Antibiotics may be used not only for the treatment of animals with a manifest clinical disease, but also for metaphylaxis, prophylaxis, growth promotion, or even in plant agriculture. Antimicrobials may also be added to feed and food as preservatives to control foodborne bacteria, inhibit spoilage microorganisms, and extend the shelf life of the final products. Decontaminants can be used to inactivate, or inhibit, the growth of pathogenic and spoilage microorganisms in fresh food, while disinfectants are mostly used to reduce the level of microorganisms in abiotic surfaces, equipment, and others [4,5].

**Figure 1:**
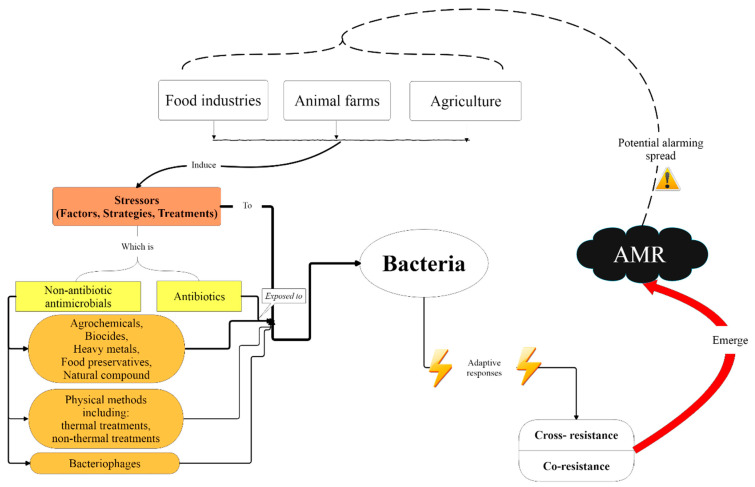
Schematic overview of factors contributing to bacterial adaptative responses. AMR: antimicrobial resistance.

Overall, foodborne bacteria are subject to several stresses during their lifecycle, and throughout all the processes associated with food production, processing and storage. These stresses can be physical, chemical, or biological, and ultimately may lead to a stress adaptation (Figure 1). The adaptative or protective response may, in turn, confer protection to the same stress or against a different type of stress, known as stress cross-adaptation [6]. Usually, this adaptation occurs as a cellular response of the bacterium to the stressor, by regulating molecular mechanisms that, ultimately, may result in the cellular repair or damage tolerance, in the maintenance of cell homeostasis, or even in the removal of the stressor [6,7]. In turn, this cross-adaptation may select variants with increased tolerance or resistance, including decreased susceptibility to several antibiotics, namely, some antibiotics relevant to clinical practice.

Taking this into consideration, this review focused on the cross-adaptation due to the non-antibiotic, food-chain-related stresses, associated with a diminished susceptibility to antibiotics and facilitation of antimicrobial emergence, and, thus, the spread of antibiotic resistance along the food chain, while also acting as an AMR reservoir.

## 2. Interaction of the Use of Non-Antibiotic Antimicrobials with A Potential Antibiotic Decreased Susceptibility

A further driver of resistance is the non-antibiotic antibacterial, such as agrochemicals, biocides, heavy metals, or food preservatives, for which bacteria could acquire antibiotic resistance by co- or cross-resistance mechanisms.

### 2.1. Agrochemicals

Agricultural practices can be vastly affected by plant diseases, which may be managed by using agrochemicals to ensure a sustainable and prolific agricultural system. However, the intensive use of these chemicals contributes to its persistence and dispersion in the environment, adversely affecting humans and the ecosystem [8,9]. Furthermore, the presence of a diversity of drugs, commonly used in humans and animals, as well as on agricultural procedures, in surface and wastewaters, even in trace amounts, may enter the food chain and potentiate the development of resistant foodborne pathogens. Some agrochemicals are ubiquitous in the food supply, and while they have been tested for human toxicity, their effect on microorganisms is not well-documented. Nonetheless, some studies address the increase in antibiotic/antimicrobial resistance caused by low levels of some chemicals (Table 1). Kleiner et al. (2007) tested the effect of adaptation of *Staphylococcus aureus* (*S. aureus*), as an indicator organism, with five compounds representing the major groups of pesticides: chlorinated hydrocarbons, carbamates, organophosphates, herbicides, and fungicides. They found that the exposure to organophosphates led to substantive changes in the minimum inhibitory concentration (MIC) values of the antibiotics tested (ampicillin, erythromycin, gentamicin, kanamycin, neomycin, norfloxacin, oxacillin, sulfathiazole, tetracycline, and vancomycin). Additionally, among four veterinary products used, amprolium, arsanilic acid, ivermectin and levamisole, amprolium, which possessed some antibacterial activity, gave rise to increased MICs. No increase was driven by arsanilic acid, a growth-stimulant feed-additive exhibiting some antibacterial activity. When agrochemical combinations were applied, a major increase in MICs to all tested antibiotics was presented [10]. The potentiation of decreased susceptibility to antibiotics by exposure to a mixture of pesticides or pesticides and antibiotics has been reported by other authors [11].

The sublethal exposure of *Escherichia coli* (*E. coli*) and *Salmonella enterica* serovar Typhimurium to commercial formulations of three herbicide (dicamba (Kamba), 2,4-dichlorophenoxyacetic acid (2,4-D), and glyphosate (Roundup)) was found to induce a change in the response to antibiotics, with MICs of antibiotics of five different classes changing up to 6-fold. A significant role of the efflux activity in the increase in tolerance of *E. coli* to chloramphenicol in the presence of dicamba, and kanamycin in the presence of glyphosate, was confirmed using an efflux pump inhibitor. In fact, the induced response to the various herbicides varied according to the exposed species, and the tolerance to different antibiotics changed with the exposure to different herbicides. The authors found that the concentration of herbicide required to induce a maximal response to an antibiotic was above the maximum residue limits allowed under international trading laws. However, a detectable antibiotic response was induced with an herbicide concentration lower than that specified in the label, and the response was additive when chemicals that cause similar phenotypic changes were combined [12]. Glyphosate-based herbicides are amongst the most heavily applied herbicides in the world. This group of compounds and their metabolites are known to contaminate the environment, especially agricultural regions; to be widely present in the food chain and be harmful to humans [41]. Due to its wide application in farms, several studies have focused on the study of changes in the susceptibility of bacteria by exposure to these compounds. In fact, historical isolates of *S*. *enterica* from a period before the introduction of the glyphosate-based herbicides presented lower MICs for these compounds than isolates collected after the introduction of the herbicide [42]. *S*. *enterica* presented a slow adaptation dynamic; however, glyphosate-based herbicide resistance has the potential to become fixed in isolates, with mutations arising close to or at the glyphosate molecular target, as well as in genes associated with stress response and tolerance [43]. Further, the exposure to glyphosate induced metabolic starvation by the down-regulation of genes associated with carbon and amino acid transport and metabolism, energy drain by the down-regulation of energy production- and conversion-associated genes, as well as other off-target effects associated with the up-regulation of flagellar and chemotaxis genes, or envelope and stress response proteins [43,44]. Although glyphosate-based herbicides may induce a vast cellular response, the observed adaptive resistance has been associated with modifications of efflux or influx [12,43,44,45]. The transient exposure of pathogenic *S. enterica* strains to sub-inhibitory concentrations of the herbicide elicited a tolerance response at the cellular level and up-regulation of the AcrAB-TolC efflux system. In turn, a chronic exposure led to the selection of glyphosate-based herbicide resistance, with no effect on cross-tolerance and cross-resistance to antibiotics [43].

Other chemical stressors, such as pesticides, have also been shown to influence the expression of genes coding for efflux or influx proteins. Consequently, environmental contamination with pesticides may be associated with antibiotic resistance. One example of this influence is pentachlorophenol, an organochlorine pesticide, which has been banned or restricted in many countries worldwide due to its environmental- and human-health-adverse effects. However, it was used for many years as a bactericide, fungicide, herbicide, defoliant, and wood preservative, and can still be detected in food products [46,47]. This compound was shown to upregulate genes coding for multidrug efflux pumps, including MexAB-OprM, an efflux pump responsible for resistance to a wide variety of antibiotics in *Pseudomonas aeruginosa* (*P. aeruginosa*) [48].

A common scenario found in the environment is the co-existence of antibiotics and pesticides. Recently, this fact was considered in the study of the effects of long-term exposure of *E. coli* K-12, as a model strain, to a mixture of pesticides on their environmental concentrations, on the development of antibiotic resistance. The authors showed that the emergence of stronger streptomycin resistance was stimulated, while the same did not occur for other antibiotics. However, when *E. coli* was exposed to the mixture of pesticides and a sub-inhibitory concentration of ampicillin, a cross-resistance with this and other antibiotics (i.e., ciprofloxacin, chloramphenicol, and tetracycline) was detected. In the case of the first scenario, the higher resistance to streptomycin was attributed to mutations associated with the antibiotic target by exposure to the pesticides. In the second scenario, diverse genetic mutations emerged from exposure to both pesticides and ampicillin, with the ones differing from the mutants resulting from ampicillin exposure, which were transcriptional-level associated. These mutations suggest that higher resistance may be gained by augmented biofilm formation, heat shock, oxidative stress or carbon starvation defenses, and the deactivation of prophage related genes. The authors proposed that the co-occurrence of pesticides and sub-inhibitory concentrations of antibiotics select, de novo, antibiotic-resistant mutants from a susceptible population in a synergistic way, leading to a higher resistance than from mutants, selected by only the antibiotic [13]. This co-existence scenario should be further explored.

Beyond the role of the adaptative response triggered by exposure to agrochemicals leading to changes in gene expression, often associated with efflux and permeability or phenotypical resistance, the acquisition of spontaneous mutations has also been related to a decrease in susceptibility to antibiotics, and attention must be given to co-resistance occurrence.

Overall, these works pointed to the significance that low-level chemical contaminations may have in decreasing the susceptibility to antibiotics, even at legal food residue levels, while highlighting the need for further study.

### 2.2. Biocides

Another important factor involved in the development of antibiotic resistance is the extensive and widespread use of biocides in the food and health system and animal facilities. These antimicrobial chemicals, including disinfectants, antiseptics and preservatives, are even more used than antibiotics. Therefore, there are large amounts of residuals of these compounds in the sewage, water from agricultural areas and other similar aquatic environments. At an appropriate level, they have an important role in the killing of bacteria, but, at the sub-MIC level, they could induce bacteria to develop resistance properties. Sometimes, co- or cross-selection with other biocides or antibiotics can also occur [21,49,50]. Several researchers proposed that some biocides can provide cross-resistance properties to antibiotics, including: triclosan; chlorhexidine; hypochlorite; chlorine dioxide; quaternary ammonium compounds (QACs); parabens; phenols; glutaraldehyde; acid anionics; peroxygen compounds (Table 1). Among them, triclosan, QACs, and chlorhexidine are widely used in more than 1000 products, from the first step of the farm operations to the final step of the food chain, and play a major role in the development of antibiotic resistance [49,51,52,53,54].

Although many biocides have different targets than antibiotics, bacteria may use the same resistance mechanisms for both biocides and antibiotics. These mechanisms include the reduction in the cell membrane and lipopolysaccharide (LPS) layer permeability, reduction in the activity of the porin channels, the overexpression of efflux pumps, or even biofilm formation [49,50,55,56,57]. The induction of mutations or SOS response and increase in the rate of gene transfer are other mechanisms that are affected by the use of disinfectants [58,59]. Studies showed that when *Salmonella* spp. variants were exposed to sub-MIC of QACs or triclosan, a reduction in outer membrane porins and increase in the expression of the AcrAB-TolC efflux pump occurred. As a consequence, these QACs- and triclosan-resistant strains presented two- to eight-fold increased MIC values for ciprofloxacin, chloramphenicol, tetracycline, and ampicillin [15,60]. In this regard, the increase in the MIC values for some antibiotics can even pass the clinical breakpoint and, if it occurs, it will be worrisome. Sonbol et al. (2019) showed that, when 78 *E. coli* isolates were treated with incremental increased sublethal concentrations of triclosan, the percentage of resistant strains increased for several main classes of antibiotics [16]. Triclosan stimulates the expression of Resistance Nodulation Cell Division (RND) family efflux pumps, such as the AcrAB-TolC in *E. coli* and *Salmonella* spp., the CmeABC and CmeDEF in *Campylobacter jejuni*, SmeDEF efflux pump in *Stenotrophomonas maltophilia*, and several of the Mex systems in *P. aeruginosa*. It also triggers the production of β-lactamase AmpC by the induction of mutations in key genes such as *frdD*, *marR*, *acrR* and *soxR*, and the activation of several systems. Therefore, the widespread use of triclosan can lead to the emergence of resistance in several foodborne pathogens [50,51,61,62,63]. Amongst Gram-positive bacteria, *S. aureus* mutants selected with QACs showed an increased *norA* expression and a cross-resistance with fluoroquinolones [17]. Further, benzalkonium chloride (classified as a QACs) could trigger the expression levels of MdrL efflux pump in *Listeria monocytogenes* (*L. monocytogenes*), leading to a decrease in the susceptibility to ciprofloxacin by increasing two-fold the MIC value, or even changing the profile from susceptible to resistant to various strains for cefotaxime and cephalothin [7,18]. In *P. aeruginosa*, the exposure to sub-MIC levels of benzalkonium chloride and chlorhexidine could decrease the expression of the repressor gene *mexR* and, in turn, induce the expression of MexAB-OprM and MexCD-OprJ efflux pumps [19,20]. In fact, the exposure to sub-inhibitory concentrations of benzalkonium chloride caused a change in the categorization of *P. aeruginosa*, from ciprofloxacin-susceptible to -resistant [19]. Further, when a group of 50 isolates of antibiotic-susceptible *Pseudomonas* was exposed to sub-inhibitory concentrations of didecyldimonium chloride and sodium hypochlorite, a significant increase was observed in the MICs of different classes of antibiotics. After incubation with sodium hypochlorite, from 41 to 92% of the isolates showed an MIC increase of at least two-fold for colistin, ceftazidime, amikacin, meropenem, gentamicin, piperacillin-tazobactam, and ciprofloxacin. Moreover, when exposed to didecyldimonium chloride, an MIC raise occurred in all the mentioned antibiotics, although for a lower percentage of isolates. Mainly by exposure to sodium hypochlorite, a resistance phenotype arises for some of the isolates [21]. In the case of *Klebsiella pneumoniae* (*K. pneumoniae*), chlorhexidine can upregulate the *smvA* gene, coding for an MFS efflux pump due to mutations in its putative repressor gene (*smvR*). Furthermore, the up-regulation of the operon *pmrK*, which plays a role in the modification of LPS, was observed; both changes led to colistin-resistant strains [22].

Additionally, some oxidizer components, such as hydrogen peroxide and hypochlorous acid, which are used widely in the poultry industry at present, can trigger oxyR radical defense or soxRS systems in bacteria. These activations might lead to an overexpression of efflux pumps that render resistance to streptomycin and rifampin [49].

Chlorine is one of the most widely used disinfection methods, mainly due to its low cost and durability. By applying chlorine to bacteria, the first attack is directed towards proteins and peptidoglycan. After this, it can enter cells primarily through diffusion and transport, penetrate the cell wall and reach the cytoplasm, and then react with cellular components and coagulate enzymes and nucleic acids, causing microbial death [64].

As found for other compounds, the adaptation of bacteria to chlorine could constitute a potential threat to food safety by inducing cross-protection to clinically important antibiotics, as well as by contributing to the spread of resistance to other bacteria [24,65]. Potenski et al. (2003) exposed five different *Salmonella* Enteritidis isolates to a single dose of chlorine and the surviving, selected mutants exhibited a decreased susceptibility to tetracycline, nalidixic acid and chloramphenicol when compared to wild-type isolates. The exposure to 25 ppm of chlorine induced the *marRAB* operon, suggesting that *mar* mutation was responsible for these higher resistances, as a global regulator controlling intrinsic resistance towards structurally and functionally unrelated antibiotics and associated with an increased expression of efflux pumps [23]. Obe et al. (2018) investigated the behavior of *S. enterica* serovar Heidelberg, exposed to sublethal chlorine concentrations. This strain changed the morphology to the rugose variant and demonstrated the ability to acquire resistance. The adapted rugose strain exhibited a reduction in susceptibility to gentamicin, streptomycin, ampicillin and ciprofloxacin, while, for the smoothly adapted strain, it was observed against sulphamethoxazole/trimethoprim and streptomycin. The authors speculated that chlorine might confer a cross-protection that aids in the survival of the tolerant population to other environmental stresses and becomes resistant to certain treatments, scenarios that could represent a potential public health risk [24]. Templeton et al. (2009) investigated the effect of chlorination on *E. coli* strains which were resistant to ampicillin and trimethoprim compared to sensitive *E. coli* strain isolated from wastewater. Only the trimethoprim-resistant *E. coli* strain was found to be more resistant to chlorine than the antibiotic-sensitive isolate, although authors reported that this difference would not be important under normal chlorination conditions, when applied in practice [25]. Huang et al. (2013) reported that chlorination treatment would only increase the risk of selection for highly tetracycline-resistant *E. coli* if high doses (>1.0 mg Cl_2_/L) were applied [26]. Similarly, the ampicillin resistance of *E. coli* strains increased by chlorination at 2 mg/L, even if chlorination did not result in an efficient disinfection method for disrupting antibiotic resistance genes (ARGs) [27]. By contrast, in the study of Rizzo et al. (2013) the chlorination process for 120 min at 2 mg Cl_2_/L did not affect *E. coli* strains’ resistance to antibiotics [66]. Venieri et al. (2016) observed different effects of chlorine treatment (at both 1 and 5 mg/L) on the antibiotic resistance profile in residual *K. pneumoniae* strains, namely, an increase in resistance to ampicillin as well as an increase in susceptibility to cefaclor and tetracycline were reported [28]. Hou et al. (2019) investigated the chlorine injury in *P. aeruginosa* at different exposure levels, and only strains with final chlorine concentrations of 4 and 8 mg enhanced antibiotic resistance against ceftazidime, chloramphenicol and ampicillin, similarly to MIC values reported by Nasr et al. (2018) against amikacin and gentamicin for antibiotic-susceptible *P. aeruginosa* isolates exposed to sub-inhibitory concentrations of chlorine disinfectants [21,29].

Furthermore, besides these selection mechanisms, exposure to sublethal concentrations of biocides can influence the horizontal transfer rate of ARGs. Jin et al. (2020) found that *E. coli, Salmonella* Aberdeen, *P. aeruginosa* and *Enterococcus faecalis* (*E. faecalis*) showed diverse resistance to sodium hypochlorite and chlorine disinfection naturally accelerated the genetic exchange in or across bacterial genera by promoting the horizontal transfer of plasmids by natural transformation. This resulted in the exchange of ARGs across bacterial genera and the emergence of new antibiotic-resistant bacteria (ARB), as well as the transfer of chlorine-injured opportunistic pathogens from non-ARB to ARB [67]. Additionally, for other biocides, Jutkina et al. (2018) showed that both triclosan (0.1 mg/L) and chlorhexidine (24.4 μg/L) can promote the horizontal transfer of antibiotic resistance [59]. Zhang et al. (2017) observed similar results and proposed that the sub-MIC of chlorine, chloramine and hydrogen peroxide could promote ARG transfer between *E. coli* strains, as well as from *E. coli* to *S.* Typhimurium, by an average of one to five-fold. They proposed that intracellular reactive oxygen species formation, SOS response, and the overexpression of conjugation-relevant genes, including *korA*, *korB*, *trbA*, *trbBp*, *traF*, *trfAp*, and *traJ*, or outer membrane-encoding genes, including *ompA*, *ompF*, and *ompC*, or oxidative stress regulatory genes, including *rpoS*, may be involved in this potentiation [58]. In general, all these findings emphasize the need for a correct and principled use of disinfectants. Therefore, a strategy to correctly use these chemicals according to their effect on antibiotic resistance or replace them by applying other techniques, should be defined.

### 2.3. Heavy Metals

In nature, some metals are essential for various physiological functions of living organisms, and others, defined as non-bio-essential metals, have no biological functions and can cause toxicity even at very low concentrations [68]. Toxic heavy metals are widely spread in the environment, naturally or by anthropogenic and zootechnical contamination. Once in the environment, their persistence, as well as their bioaccumulation in the food chain, have long-term impacts, both posing food safety issues and acting as a selective pressure for adaptations in microbial communities, even considering that heavy metals are more stable and resistant to degradation than antibiotics [30]. Metal resistance is a common phenotype in many microorganisms and, considering that resistance is achieved by similar strategies to those of antibiotics, it is clear that metal resistance can contribute to the emergence and spread of antibiotic-resistant strains. The earliest report of this acquired metal resistance dated back to 1960 in a mercury-resistant *S. aureus* strain [69]. Mechanisms of co-selection, by both co-resistance and cross-resistance, are involved in the evolution of multiple antibiotic-resistant pathogens, and it is an evolving danger to human health due to the development of metal-driven, multiple-antibiotic-resistant pathogens. Imran et al. (2019) described metal and antibiotic pollutants in terrestrial and aquatic environments and their possible role in the evolution of multiple antibiotic-resistant human pathogens through the co-selection mechanism, achieved by reduced membrane permeability, inactivation, efflux pump, and the mutation of gene encoding targets of both antibiotics and metals, as well as the extracellular sequestration of antibiotics and biofilm formation. Their review summarizes that the reduction in membrane permeability is associated with resistance to arsenic, manganese, cobalt, copper, silver, zinc, ciprofloxacin, β-lactams, chloramphenicol, and tetracycline, and that the antibiotic/metal inactivation is associated with arsenic, mercury, chloramphenicol and β-lactams resistance, as well as the fact that the rapid efflux of metal or antibiotic is associated with arsenic, cadmium, cobalt, copper, zinc, tetracycline, chloramphenicol and β-lactams. In addition, on one hand, the alteration in the metal/antibiotic target is associated with copper, mercury, zinc, ciprofloxacin, rifampicin, β-lactams, and trimethoprim, and on the other, the maintenance of multi-resistance is performed by plasmids harboring antibiotic resistance genes, as well as genes encoding resistance to heavy metal, for example, conjugative plasmids for the co-transfer of macrolide and glycopeptide copper resistance, or for genes encoding β-lactamases (*bla*CTX-M) or even *bla*, *str*AB, *cat*I, *sul*II and *dhfr*1b genes for multiple resistance to ampicillin, streptomycin, chloramphenicol, sulphonamide and trimethoprim, as well as the linkage of copper, silver and mercury-resistance operons. Furthermore, class I integrons carrying genes encoding for the multidrug resistance efflux pump *czc*A possess genes responsible for the extrusion of zinc, cadmium, and cobalt ions, and the heavy metal efflux pumps (AcrAB, AcrD, CzcCBA, MdtABC and TetA) are responsible for resistance towards heavy metals, metalloids, and antibiotics. All the reported mechanisms of co-selection resulted in an upcoming risk to human health due to the development of metal-driven, multiple-antibiotic-resistant bacterial pathogens [70]. Nguyen et al. (2019) reviewed the association between ten heavy metals (cadmium, copper, zinc, mercury, cobalt, chromium, nickel, silver, iron, and arsenic) and antibiotic resistance versus different antibiotic classes within human pathogenic bacteria (*P. aeruginosa, E. coli, Klebsiella* spp., *Salmonella* spp., *Enterobacter* spp., *E. faecium, Citrobacter* spp., *Proteus* spp., *Acinetobacter baumannii (A. baumannii), Serratia* spp., *S. aureus* and *Shigella* spp.) in water, wastewater and soil. The authors showed that heavy metals are important co-selecting agents in the proliferation of antibiotic resistance in priority human pathogens. Zinc and cadmium resulted in the most observed heavy metals associated with resistance to antibiotics in all environments. *P. aeruginosa* and *E. coli* were the most investigated bacteria and were the species with the highest reported co-occurrence of resistance to several heavy metal and antibiotic classes. It should be underlined that, in soil, Gram-negative bacteria yielded higher levels of resistance to both heavy metals and antibiotics compared to Gram-positive bacteria [30]. Squadrone et al. (2020) considered the presence of co-resistance to metals and antibiotics in aquatic environments worldwide and stated that, even in environments where antibiotics have never been used, the presence of heavy metals is sufficient to select for antibiotic-resistant bacteria and antibiotic resistance genes. In addition, a similar association of co-resistance to heavy metals and antibiotics (ampicillin and nickel, penicillin and copper, erythromycin and cadmium) in bacteria isolated from very distant water environments was reported. Therefore, the pressure exercised by a specific heavy metal seems to be selective to a particular antibiotic, and potentially transferable by horizontal gene transfer to other pathogenic bacteria [68]. For a more detailed understanding of all the associations of heavy metals with antibiotic resistance in the different microorganisms investigated in the literature, the authors suggest referring to the above-mentioned reviews. However, despite the extensive literature on this issue, some drawbacks have to be noticed, namely the methodological gap in terms of standardized MICs and minimum selective concentrations, and the lack of information regarding the patterns of exposure (time and concentrations) leading to the co-selection of bacteria resistant to heavy metals and antibiotics. Further, the integration of culture-based and molecular-based methods, or even metagenomic approaches and sequencing technologies able to identify standardized methods and provide new insights into mechanisms explaining the variability of these associations, are still lacking.

### 2.4. Food Preservatives and Decontaminants

Antimicrobials are widely used throughout the food chain, whether in the agriculture and livestock industry or directly in foods, with the direct or indirect potential to affect the development of antimicrobial resistance by bacteria dispersal along the farm-to -fork continuum. While the adaptation to antibiotics and biocides has been widely studied, the response and adaptation of foodborne bacteria to food preservatives and decontaminants is poorly studied (Table 1.).

Acids are included in the compounds more vastly used in food preservation and decontamination procedures. Acidic stress is as a primary barrier in food and, therefore, the adaptive responses of foodborne pathogens may enhance resistance to other substances and could lead to cross-protection. When exposed to acid stress, microorganisms may adapt to these environments by the overexpression of sigma factor, synthesis of outer membrane proteins, by alteration of the membrane permeability and of the cell membrane fluidity by modifying the membrane lipid composition. These changes have a direct effect on the influx and efflux of antibiotics across the cell membrane and thus on the antibiotic susceptibility of the bacterial cell itself [31].

*Salmonella* spp. is a crucial foodborne pathogen faced with different stress conditions, both in their environmental niches, mainly during the production and storage of foodstuff, as well as in the defensive barriers (gastrointestinal tract) of their hosts. As a result, the adaptive response of *Salmonella* to a range of biotic and abiotic stresses is well known, starting from the acid tolerance response of *S*. Typhimurium. Four wild-type strains of *S.* Typhimurium, *E. coli*, and *S. aureus*, under conditions of low-pH stress of 5.0, 4.5, or 4.0 obtained by acidification with HCl, significantly altered their antibiotic resistance levels, with the higher MIC of amikacin, ceftriaxone and nalidixic acid than controls. This decreased susceptibility was maintained after the stress was removed in *E. coli* and *S. aureus*, but not in *S.* Typhimurium, suggesting that the observed increase in MICs could be a stress response involving the whole population or the selection and outgrowth of a subpopulation of hyper-resistant clones [32]. In contrast, Bacon et al. (2003) and Hughes et al. (2010) found no association between antibiotic resistance and acid stress response for *Salmonella* spp. isolates [71,72].

Even in *L. monocytogenes*, which is a versatile organism with the ability to survive for long periods under adverse environmental conditions including cold storage, high NaCl concentrations and acidic pH, some adaptive responses were evidenced. Al-Nabulsi et al. (2015) exposed three *L. monocytogenes* strains to three different levels of pH (6.0, 5.5, and 5.0) and the acid-stressed cells at pH 5 resulted as less susceptible to streptomycin, gentamicin, ampicillin, penicillin, ciprofloxacin and enrofloxacin; this profile was maintained for at least a day after the stress was removed [33]. Similarly, but at lower pH, the study of Komora et al. (2017) investigated the ability of antibiotic-susceptible and -resistant and multi-antibiotic resistant *L. monocytogenes* strains from food and clinical origin to survive acidic stress (1% lactic acid, pH 3.5). The results demonstrated that food strains resistant to ciprofloxacin, nitrofurantoin and erythromycin were significantly more resistant to acidic stress than susceptible strains [34]. In contrast, Faezi-Ghasemi and Kazemi (2014) observed an increase in the susceptibility of *L. monocytogenes* subjected to the sublethal level of the environmental stresses (HCl, pH = 5.0) with a decreased MIC for gentamicin and rifampicin [73]. For *A. baumannii*, Ebinesh et al. (2018) tested the influence of pH stress (acid at 3, 5, and 6 and alkaline at 9 and 10 pH for 2 h) on the antibiotic susceptibility of six strains; the exposure to acidity conferred a higher degree of resistance than alkalinity to amikacin, piperacillin, tazobactam, imipenem and meropenem. However, only norfloxacin presented a significantly different result [35]. In five strains of *Cronobacter sakazakii (C. sakazakii*), the effect of extreme pH (3.5 for 30 min) stress was evaluated by Al-Nabulsi et al. (2011) and was shown to increase sensitivity toward streptomycin, gentamicin, kanamycin and doxycycline, while augmenting resistance to tetracycline, tilmicosin, florfenicol, amoxicillin, ampicillin, vancomycin and enrofloxacin (with variable strain response toward ciprofloxacin). It is notable that some isolates did not have the same behavior within the considered populations. The uniformly increased resistance of acid-stressed strains to antibiotics could be explained by alterations in the cell envelope, including reduced porin synthesis, changes in lipopolysaccharides, over-expression of multi-gene components or operons and the alteration of antibiotic target sites [31].

Considering the diverse results obtained and a practical application of acidic stress in food processing, the use of lactic and acetic acids for decontamination of food products was recently addressed in a scientific opinion on the evaluation of the safety and efficacy of these acids on the reduction in microbiological surface contamination on pork carcasses and pork cuts, conducted by the European Food Safety Authority Panel on Food Contact Materials, Enzymes and Processing Aids by request of the European Commission. This report considered several points, namely the potential selection and emergence of bacteria with reduced susceptibility to biocides and/or resistance to antibiotics with therapeutic use. The existence of insufficient indications that could support the hypothesis that a sublethal exposure of bacteria to organic acids may promote or augment antibiotic or biocide resistance was pointed out [74]. Despite the fact that a possible acid adaptation may occur, leading to a reduced efficacy of organic acids or other chemical treatments [75,76] (and reviewed by [77]), or even to a small decrease in susceptibility to some antibiotics [23,32], their impact on public health is unknown [74].

Besides acids, other food preservatives have been studied. Potenski et al. (2006) explored the potential of widely used food preservatives to confer antibiotic resistance to *Salmonella* Enteritidis, showing that a single exposure to acetic acid, sodium benzoate, or sodium nitrite can result in a stable decrease in susceptibility to tetracycline. Although clinical resistance breakpoint was not reached, the MIC values were augmented up to four-fold, with data suggesting an association with *mar* mutation [23]. Alonso-Calleja et al. (2015) studied the adaptation and cross-adaptation of *E. coli* ATCC 12806 by passage through gradually higher concentrations of trisodium phosphate, sodium nitrite, and sodium hypochlorite, observing an adaptative tolerance to these compounds. This adaptation was stable for sodium nitrite and sodium hypochlorite and could be at least partially caused by efflux pumps and changes in cell surface hydrophobicity [78]. Moreover, strains exposed to these biocides displayed a stable reduced susceptibility to various antibiotics, with sodium nitrite, causing a change from the category of susceptible to resistant for 14 out of the 29 antibiotics tested (mainly aminoglycosides, cephalosporins and quinolones). In addition, the adaptation to sodium nitrite and sodium hypochlorite substantially improved biofilm formation ability. From the tested compounds, trisodium phosphate showed positive effects when considering the context of food safety, with cells adapted and/or exposed to sub-inhibitory concentrations of trisodium phosphate showing a transient tolerance to these biocides, less tolerance to antibiotics, and little potential to form biofilms [14]. Moreover, EDTA, which, at low concentrations, is widely used as a food preservative, was shown to decrease EfrAB efflux system expression in *E. faecalis* and *Enterococcus faecium* (*E. faecium*), at 3 mM of EDTA, reducing the MIC of various antibiotics or biocides [79]. Multidrug resistance of enterococci, a ubiquitous bacterium that can be frequently found in foods as a contaminant, was reported and related to intrinsic or acquired mechanisms, and often associated with the over-expression of efflux pumps, pointing to EDTA as a good choice as a food preservative, preventing the spread of multidrug-resistant enterococci throughout the food chain [79].

Other approaches have highlighted the co-resistance between chemical preservatives or physicochemical treatments applied at different stages of the food chain and the antibiotic or biocide resistance profile of bacteria. Romero et al. (2017), when studying several bacterial strains isolated from seafood, found that a high percentage of the isolates studied (75.86%) were resistant to at least one antibiotic or one biocide, and 6.90% were resistant to at least three biocides and at least three antibiotics. Beyond the moderate or strong positive correlations of tolerance detected amongst biocides, antibiotics and between antibiotics with biocides and other antimicrobials, a noteworthy moderate positive correlation was found for sodium lactate and trisodium phosphate with ampicillin and imipenem, but not with biocides [80]. Despite the lack of correlation between the tolerance to chemical preservatives and biocides, Gadea et al. (2017) described that triclosan-adapted Gram-positive and Gram-negative bacteria show a generalized increase in tolerance to preservatives (sodium nitrite, sodium nitrate, potassium sorbate, sodium propionate and 4-hydroxybenzoic acid). On the other hand, strains adapted to QACs, hexachlorophene [2, 2′-methylenebis (3, 4, 6-trichlorophenol)] or chlorhexidine presented a generalized increase in the susceptibility to preservatives, with some exceptions, which may be strain-dependent [81]. A correlation between antibiotic resistance and an increased tolerance to stress was described for drug- or multidrug-resistant *S. aureus* strains or antibiotic-adapted *S.* Typhimurium. Resistant strains presented similar or higher survival rates than the susceptible ones to environmental stresses typically found in the food chain (acidic, heat and osmotic stresses) [82,83]. A similar relation was established for various *Salmonella* serotypes, where a correlation between survival to acidified sodium chloride and trisodium phosphate was presented. Antibiotic-sensitive strains were shown to be more susceptible to both compounds when compared with multi-resistant ones. The significance of the correlation was more evident for acidified sodium chloride. When evaluating citric acid, no correlation was found [84]. Additionally, a possible cross-protection of antibiotic-resistant isolates of *L. monocytogenes* to food-associated stresses was reported. *L. monocytogenes* strains resistant to various antibiotics (e.g., ciprofloxacin, nitrofurantoin and erythromycin) were shown to be more resistant to the application of osmotic and acidic stress than susceptible strains [34].

Similar to other stressors, antibiotic resistance could be additionally potentiated in an indirect form by environmental stresses, such as the ones found in food preservation systems, such as high/low temperature, pH and osmotic stresses, which may increase the rates of horizontal transmission of plasmids between *E. coli* strains or *E. coli* and *S.* Typhimurium, stimulating the antibiotic resistance exchange. Thus, the use of sublethal food preservation systems may contribute to the dissemination of antibiotic resistance [85]. For example, the rate of horizontal transmission of antibiotic resistance plasmids among *E. coli* strains and between *E. coli* and *S.* Typhimurium was investigated by McMahon et al. (2007), showing an increased rate of horizontal transmission of plasmids R386 and TP307 when a prestressed donor and recipient cells are mated under sublethal acid stress [85].

### 2.5. Natural Compounds

A growing interest in natural compounds as an alternative to chemical disinfectants and food preservatives was reported, focused on their safety and ability to act in multiple-cell targets, thus potentially limiting the development of resistance [86]. Furthermore, to reduce the use of agrochemicals, plant extracts were used as biopesticides, with some already-commercialized organic pesticides based on essential oils (reviewed by Durán-Lara et al. in 2020) [87]. Nonetheless, caution must be taken in the potential adaptation of bacteria to these molecules [88]. This phenomenon has been observed for several antimicrobials, and some studies have shown that the exposure of bacteria to sub-inhibitory concentrations of natural compounds may lead to the cross-resistance to antibiotics or even food-associated stresses, while others have pointed out that the use of natural compounds would not influence the resistance to antibiotics. Considering this, when evaluating the antimicrobial potential of natural compounds as food preservatives or for application in several stages of the food chain, the possible facilitation of the emergence of AMR by microbial exposure should be assessed. Various works have presented this approach, evaluating the possible development of tolerance/resistance to antibiotics by the application of these compounds as sublethal stresses.

For example, epigallocathechin gallate (EGCG), a major polyphenolic component of green tea extract, is known to possess many beneficial properties, including antibacterial activity. However, a short exposure of staphylococci strains to a sublethal concentration of EGCG led to an adaptive response toward EGCG and a decrease in susceptibility to vancomycin and oxacillin, and even ampicillin. This cross-protection was associated with an increased cell wall thickness, and so an enhanced tolerance to cell-wall-targeting antibiotics [36]. Furthermore, it was associated with an induction of a cell wall stress response in *S. aureus*, modulated by the two-component VraSR system, in the same manner as cell-wall-active antibiotics [37]. The upregulation of genes encoding efflux system proteins by the exposure of *Pseudomonas fluorescens*, a spoilage bacterium commonly found in diverse food matrixes, to EGCG, has been described. Efflux is one of the mechanisms commonly associated with cross-resistance to compounds without a chemical relation, and even the selection or acquisition of other resistance mechanisms [89], which point to a possible cross-resistance with antibiotics and the facilitation of AMR emergence.

The cross-adaptation induced by other natural products has been studied; this is the case of the tea tree oil (*Melaleuca alternifolia*), an essential oil which is widely available and vastly investigated as an alternative antimicrobial, anti-inflammatory and anti-cancer agent for topical use, since it is toxic if ingested in high doses [90,91]. Nonetheless, it has been suggested as a natural food preservative [92], and several authors analyzed the potential for the cross-adaptation of and, reduction in the susceptibility of, some foodborne pathogens to antibiotics. The effect of a sublethal challenge with tea tree oil on the antibiotic resistance profiles of *E. coli*, *S. aureus*/methicillin-resistant *Staphylococcus aureus* (MRSA), *S.* Typhimurium and *S.* Enteritidis have been studied, showing that an increase in the MIC was recorded for all organisms, against the majority of the 10 clinically relevant antibiotics tested [93]. Further, after the adaptation of staphylococci strains to tea tree oil, a reduction in the susceptibility to mupirocin, fusidic acid, chloramphenicol, linezolid, and vancomycin was observed, although this was a transient, decreased susceptibility [38]. Another study focusing on a similar analysis reported that exposure to tea tree oil did not present any global effects on the development of antibiotic resistance in *S. aureus*, *S. epidermidis*, and *E. coli*, and that the repeated exposure to its main component, terpinen-4-ol, did not lead to a decrease in susceptibility. In fact, the authors suggest that, if an adaptive response was induced, it would not alter the antimicrobial susceptibility or conferred cross-protection to other antimicrobial agents [90].

Moreover, the bacterial adaptation of *E. coli* strains to *Thymus marroccanus* essential oil, carvacrol and thymol were shown to induce a reduction in susceptibility to antibiotics. Variants selected with increased concentrations of thymol showed the highest rise in level of resistance to chloramphenicol, nalidixic acid, tetracycline, and erythromycin. The authors explored the membrane-associated mechanisms of resistance and observed an overexpression of an efflux pump immunorelated to AcrAB-tolC and a decrease in the expression of outer membrane proteins in adapted strains [94]. A diminution in the susceptibility of *Serratia marcescens* to antibiotics was also found after 50 sequential passages with a sub-inhibitory concentration of oregano essential oil, while the same was not observed for cinnamon essential oil or other bacteria, such as *Proteus mirabilis*, *P. aeruginosa*, or *Morganella morganii* [39]. Further, the selection of *E. coli* mutants that were resistant to pine oil, frequently used as a component of household products, previously showed resistance to tetracycline, ampicillin, chloramphenicol, and nalidixic acid. The cross-resistance was possibly associated with efflux system overexpression [95]. Several studies supported the idea that antibiotic resistance genes may be upregulated by natural compounds. The exposure of *S.* Enteritidis to trans-cinnamaldehyde and eugenol induced the upregulation of multiple antibiotic resistance (*mar*) locus genes by both compounds, and of efflux pump genes *acrAB* by eugenol, while the compounds downregulated the expression of outer membrane proteins [40]. Similarly, thymol and carvacrol upregulated genes encoding efflux pumps in *E. coli* O157:H7 [96]. The strains’ adaptation to natural compounds by the regulation of efflux pumps, and outer membrane proteins can be associated with an increased resistance to antibiotics, as antibiotic resistance also relies on these mechanisms.

Some antimicrobial peptides are currently used as food preservatives, such as nisin and related compounds such as pediocin, which are secreted by lactic acid bacteria [97]. Nisin is a natural antimicrobial agent, which has been widely studied for food applications, namely, as a dairy preservative. It is Generally Recognized as Safe (GRAS) by the U.S. Food and Drug administration and suggested for use as an antimicrobial agent to inhibit the outgrowth of *Clostridium botulinum* spores and toxin formation in pasteurized cheese spreads [98,99]. However, the use of nisin was described as creating nisin-resistant bacteria with a frequency of from 10^−7^ to 10^−2^ [100], and several studies reported that nisin-resistant bacteria are less susceptible to antibiotics. In fact, nisin-resistant *E. faecalis* strains were less susceptible to various antibiotics, and a decrease in susceptibility to antibiotics was observed with the gradual reduction in nisin susceptibility [101]. A similar observation was made for nisin-resistant *L. monocytogenes*, for which cross-resistance was detected to aminoglycosides: kanamycin and streptomycin, and to the membrane disturbing polymixin B [102] and for *Streptococcus bovis* to ampicillin [103].

Beyond antibiotic resistance, bacterial adaptation to natural compounds may also lead to increased resistance to commonly used biocides. In fact, a study characterizing the adaptation of *S.* Typhimurium to sublethal concentrations of thymol, carvacrol, citral, and eugenol, showed increased cell resistance to the bactericidal activity of peracetic acid and didecyl dimethyl ammonium bromide [88].

Although several works reported a cross-adaptation with natural compounds, other studies reported, that the continuous mode of use of eugenol and citral would not pose a risk of resistance for *S. aureus* or *L. monocytogenes* [104]. A similar behavior was observed for resveratrol [105]. Additionally, a short incubation with sub-inhibitory concentrations of thymoquinone, the main, biologically active component of the volatile oil of *Nigella sativa* seeds, increased the susceptibility of *C. sakazakii* to ampicillin and cefoxitin [106]. A similar trend was observed for multidrug-resistant *S. enterica* isolates from human outbreaks or from poultry origin, for which no direct-tolerance or cross-tolerance to ciprofloxacin were induced after habituation in sub-inhibitory concentrations of *Origanum vulgare* L. essential oil (½ or ¼ MIC for 24, 48, and 72 h) [107].

## 3. Physical Methods of Food Processing That May Influence Antibiotic Susceptibility

Throughout the food chain, bacteria are continually exposed to several factors, strategies and treatments that determine various chemical (acids, chlorine) and physical (heat, pressure and radiation) stresses, from primary production to food processing and preservation, as well as at-home preparation. As was clearly described in the study of Liao et al. (2020), the interplay of antibiotic resistance and stress tolerance of bacteria is crucial from a food safety perspective, because various food-processing technologies can sharpen the resistance of pathogenic bacteria in foods to a range of currently used antibiotics, and vice versa, which poses a potential risk to food safety and human health [7]. Following this, a comprehensive evaluation of the cross-resistance phenomenon associated with physical treatments was performed.

### 3.1. Thermal Treatments

Heat treatment methods are widely used in the food-processing industry to inactivate pathogenic and spoilage microorganisms and destroy enzymes, as well as to extend the shelf life of foods. Nonetheless, these methods could trigger a bacterial stress response in terms of both thermal tolerance and induce cross-adaptation for antibiotic resistance [7]. Few studies in the literature show how thermal stresses affect the selection of antibiotic-resistant bacteria or their susceptibility to antibiotics, with contrasting results. McMahon et al. (2007) exposed wild-type strains of *S.* Typhimurium, *E. coli*, and *S. aureus* at 45 °C, increasing their susceptibility to amikacin, ceftriaxone, trimethoprim; amikacin, ceftriaxone, nalidixic acid; and gentamycin, oxacillin, erythromycin, respectively. However, these changes were not permanent, probably due to an alteration in membrane fluidity that reduces the rates and efficacy of antibiotic binding and their import or export through bacterial membranes [32]. In contrast, an adaptation to environmental heat stress (at 45 °C for 2 h) of one strain of *L. monocytogenes* with increased resistance to trimethoprim-sulfamethoxazole, tetracycline, chloramphenicol, penicillin, ampicillin, gentamicin, and rifampicin was reported, with a from two- to four-fold increase in the MIC. The authors concluded that, in *L. monocytogenes*, the stress responses to heat shock induced stress proteins [73]. Rodríguez-Verdugo et al. (2013) heat-adapted *E. coli* strains for about 2000 generations at 42 °C, described mutations within the *rpo*B gene encoding the beta subunit of RNA polymerase that conferred different levels of rifampicin resistance [108]. Ebinesh et al. (2018) showed that, in six strains of exposed *A. baumannii*, during their stationary phase, to domestic environmental stress, namely, temperatures ranging from 5 to 45 °C, a significant reduction in the susceptibility occurred in all the antimicrobial tested (amikacin, norfloxacin, piperacillin, tazobactam, imipenem, and meropenem), only at 45 °C. As an exception, for norfloxacin, a significant variation in susceptibility was exhibited at both 20 and 40 °C. The higher resistance exhibited is suggested to be due to alterations in the expression of porin channels, the induction of efflux pump expression, or to the expression and synthesis of stress proteins, especially sigma factors (RpoS and SigB, respectively, for Gram-positive and Gram-negative bacteria), which could be responsible for the reduction in susceptibility to a majority of the antibiotics. It should be underlined that, in this study, no specific investigations were performed on the tested strains and the antimicrobial susceptibility tests were performed by disk diffusion method and no MIC was determined [35]. Moreover, an increased resistance of five strains of *C. sakazakii* to broad-spectrum antibiotics (streptomycin, gentamicin, kanamycin, neomycin, tetracycline, doxycycline, tilmicosin, florfenicol, ampicillin, amoxicillin, vancomycin, ciprofloxacin, and enrofloxacin) was reported under heat stress (55 °C for 5 min) by Al-Nabulsi et al. (2011). This uniform increase in resistance could be considered as a likely effect of one or more sublethal cellular injury events that affected heat shock protein synthesis, membrane receptors and the efficacy of binding proteins [31].

However, it should be mentioned that all the above-mentioned studies consider thermal stresses that could not be absolutely compared with the heat treatments routinely used in the food industry. For example, in the dairy industry, pasteurization and sterilization by Ultra-High Temperature (UHT) are thermal treatments of uncontested interest. The study of Taher et al. (2020) investigated the presence of 1 genomic and 9 plasmid-mediated AMR genes in commercial pasteurized and UHT milk samples; a high prevalence of *sul2* (67.9 and 42.6%), *tetA* (54.8 and 27.9%), *tetM* (31 and 26.5%), and *bla*TEM-1B (42.9 and 32.4%) was, respectively, detected in pasteurized and UHT milk, while *mecA* was not detected. This study resulted from a concern associated with the inability of pasteurization to destruct plasmid-mediated AMR genes and their possible horizontal transfer from pasteurized milk [109].

### 3.2. Non-Thermal Treatments

#### 3.2.1. Ultraviolet (UV) and UV-Based Advanced Oxidation Processes

Ultraviolet (UV) radiation uses physical energy, and it is a non-thermal and non-chemical technology used by the food industry for liquid and solid surface decontamination, to control foodborne pathogens and spoilage microorganisms, as well as viruses and protozoa. UV radiations at short wavelengths, in the range of from 220 to 280 nm, result in physical damage to the nucleic acids and inhibit bacterial replication by induction of the formation of cyclobutene pyrimidine dimers, which blocks DNA replication and transcription, leading to cell death [110]. However, the repair mechanism of UV damage, especially by photoreactivation, is a major disadvantage of UV disinfection [111]. The germicidal effects of UV radiation mainly depend on the UV dose (J/m2) [110] and the removal effect of UV on various ARBs, even if selective, was much stronger than that of ARGs [112]. The commonly used dosages of UV do not guarantee the complete inactivation of ARGs, for which an effective removal occurs at a much higher UV fluence (>100 to extreme values of 200−400 mJ/cm^2^), for damaging DNA to avoid transformation [66,112,113]. In addition, in case of repeated UV treatments, mainly with an insufficient dose or at (sub)lethal intensities (<10 mJ/cm^2^), the fate of ARGs and extracellular materials during and after the disinfection process is a concern regarding their insufficient control and transfer [114]. Consequently, the residual ARGs could enter pathogenic microorganisms through a transformation and transduction in the environment, posing a threat to human health, because bacterial-resistant variants could be generated [66,112]. This is a crucial issue for the food industries, in which, on the one hand, UV radiation was considered the third and fourth technology with higher commercial application, or emerged in food production [110], and on the other, exposure to lower or sublethal UV-C doses may frequently occur in selected niches (under objects or in cracks and crevices and other harborage sites or due to the presence of organic matter) or for cells within microbial biofilms [115].

In the literature, microbial exposure to other common stresses belonging to decontamination and sanitation strategies is scarce, and the results of susceptibility to different antimicrobials in several foodborne pathogens, exposed to UV treatments, were affected by the investigated microorganism, the target antibiotic, the UV exposure and the contact time. In particular, Rizzo et al. (2013) reported a decrease in the MIC values of multiple-antibiotic-resistant *E. coli* strains to ciprofloxacin, up to two-fold, after 60 and 120 min of UV irradiation, whereas no changes were observed for amoxicillin and sulfamethoxazole [116]. Pang et al. (2016) indicate that UV ampicillin-resistant *E. coli* strains became more resistant to ampicillin by irradiation at 40 mJ/cm^2^: hemi-inhibitory concentration (IC_50_) was used as an index of the distribution of ampicillin-resistant *E. coli* after exposure at a different dose of UV irradiation. There was no change in IC_50_ when the UV dose was below 10 J/m^2^, whereas it increased 1.5-fold when the UV dose reached 40 J/m^2^, increasing the risk of selection for *E. coli* strains with high ampicillin resistance [27]. In the study of Zhang et al. (2017), a selective change in the inhibition zone diameters of surviving antibiotic-resistant *E. coli* and slight damage to ARGs were reported after UV exposure at 80 mJ/cm^2^. Strains initially resistant to antibiotics were more difficult to alter than those susceptible to antibiotics because of the existence and persistence of corresponding ARGs. In fact, decreased inhibition halos were observed for ampicillin, streptomycin, gentamicin, cefotaxime, chloramphenicol, ciprofloxacin, and norfloxacin in the case of *E. coli* with low antibiotic resistance phenotype; whereas, in the case of multiple-antibiotic-resistant strains, a decreased susceptibility was shown only for gentamicin and chloramphenicol, and an increase in the halo of inhibition for norfloxacin was reported [117]. Venieri et al. (2016) observed different effects of UV treatment on the antibiotic resistance profile in residual *K. pneumoniae* strains: namely, an increase in resistance to ampicillin as well as an increase in susceptibility to cefaclor and tetracycline were reported [28]. The study of Álvarez-Molina and Colleagues (2020) investigated the ability of UV-C treatment to induce cross-resistance or co-resistance to clinically relevant antibiotics. A gradual decrease in bacterial susceptibility is reported in a total of five variants out of a total of 174 strains, namely, 3 *L. monocytogenes* (versus ciprofloxacin and erythromycin) and 2 *Salmonella* spp. (*S*. Typhimurium and *S.* Enteritidis versus streptomycin and ciprofloxacin), were exposed to repeated UV treatments. An increase in the MIC values of these resistant variants was observed, with the consequent necessity of intensifying the treatment (from 3 to 5 at day 1 and from 4 to 8 mJ/cm^2^ at day 10) to reach a similar MIC after adaptation. The genomic background of the strains seems to be important for the emergence of the variant strains: *L. monocytogenes* displayed several variants whereas, for other strains, it was not possible to identify AMR variants. In addition, the number of AMR variants was strongly dependent on the type of antibiotic tested, mostly versus antibiotics that act at the level of protein synthesis through binding to different ribosomal subunits (aminoglycosides, tetracyclines, and glycylcyclines) or DNA replication and cell division, by targeting the DNA gyrase and topoisomerases (fluoroquinolones), as well as for polymyxins which act by depolarizing the cellular envelopes. Thus, suggesting that the AMR selection may depend on the mechanism of action of the antibiotic and, therefore, on the antibiotic family. The phenotypic characterization of *Salmonella* antibiotic-resistant variants has deepened the identification of changes in the patterns of susceptibility to a wide range of antibiotics, showing different susceptibility patterns and, therefore, suggesting that a parallel divergent evolution occurred, namely, nucleotide polymorphisms. Finally, whole-genome sequencing analysis of *S*. Typhimurium showed that no mutations related to the streptomycin resistance genes were detected; however, only mutations in several stress response-associated genes (*crl, rcsC, ptsJ*, and *cytR*) were observed. *S.* Typhimurium had a Met86Ile SNP in the *gyr*A gene, which was probably responsible for its reduced susceptibility to fluoroquinolones, as well as stress-response-associated genes (*uvrB*, *zntA*). The mutations identified in genes associated with AMR or the stress response suggest that the UV-resistant variants may be generated through adaptive mutagenesis, following repeated microbial exposure to these decontamination technologies at (sub) lethal intensities. It should be highlighted that the used intensities were lower than those used in practice in food industries [115]. Li et al. (2021) reported that *P. aeruginosa* strains surviving an increase in UV irradiation dose from 5.50 to 11.0 mJ/cm^2^ presented a reduced susceptibility to tetracycline, ciprofloxacin, and polymyxin B, with MIC values increasing from 1.8-fold up to four-fold. No changes were observed for ceftazidime, chloramphenicol, or gentamicin resistances. Mechanistically, UV exposure caused oxidative stress in *P. aeruginosa*, inducing the dysregulation of genes, and contributing to the related antibiotic resistance genes, involving an elevated expression of the *mexC* gene, which encodes a protein of the MexCD-OprJ pump, which functions as a determinant of antimicrobial resistance to several clinical antimicrobials, such as ciprofloxacin and tetracycline, but not, for example, polymyxin B. The susceptibility to chloramphenicol, a known substrate of MexCD-OprJ, was not affected. The authors concluded that an insufficient UV, decreasing the physiological antibiotic susceptibility in the survival of *P. aeruginosa*, may help bacteria adapt to or survive in adverse environments, posing a potential risk by reducing bacterial antibiotic susceptibility in the environment [118].

In general, UV irradiation inactivates several ARBs and the selectivity of UV disinfection to ARB and ARGs might lead to an increase in specific ARB ratios [112,113]. Guo et al. (2013) reported that UV treatment significantly decreased erythromycin and tetracycline-resistant heterotrophic bacteria, as well as their typical ARGs, even if the removal of ARB and ARGs was not statistically correlated. In addition, tetracycline-resistant bacteria showed more tolerance to low UV fluence, owing to the fact that UV treatment has selectivity for bacterial antibiotic resistance [119]. Conversely, no significant difference in inactivation was observed between tetracycline-resistant *E. coli* and antibiotic-sensitive *E. coli* exposed to 10 mJ/cm^2^ UV treatment [25,26]. In another study, UV radiation affected *E. coli* strains resistant to ciprofloxacin, but no changes to amoxicillin and sulfamethoxazole were observed, as well as no effective control of ARB spread [116]. A transitory rather than permanent microbial inactivation with re-growth to pre-treatment levels of cultivable bacterial populations, namely, enterobacteria, enterococci, and heterotrophs, may occur after UV radiation, which can also lead to an increase in ARG prevalence [120]. Similarly, UV disinfection at 5, 10, and 20 min did not appear to be effective after 24 h of disinfection of *Aeromonas, Chryseobacterium, E. coli*, *Pseudomonas* and *Serratia* tetracycline-resistant isolates, which displayed regrowth and reactivation, with the only exception occurring in *Acinetobacter* isolates. Further, no consistent reduction was immediately observed in the concentration of the *tet*W gene fraction [121]. Although most studies were on bacterial inactivation and fewer studies focused on ARG inactivation, the findings reported that UV treatment could lead to selection for certain ARGs. This selection may result in teh potential transfer of antibiotic resistance to the environment (namely, versus *tetA*, *tetG*, *tetR*, *tetX*, *mecA*, *vanA*, *ampC*, *amp*^R^, *Kan*^R^, *sul*1, *intl*1 and *bla*T_EM-1_ genes) and a much lower inactivation rate for ARGs [113]. In closing, in recent years, a significant importance has gone into UV-based Advanced Oxidation Processes (AOPs) that combine UV treatment with other processes with various radical promoters, such as chlorine, H_2_O_2_, O_3_, and H_2_O_2_/Fe^2+^ UV (photo-Fenton) [113,122], as well as ionizing radiation, including electron beam and gamma radiation [123]. AOPs seems to be more promising in terms of both the inactivation of resistant bacteria and their genetic material compared to conventional disinfection methods, such as chlorination or UV-C radiation, due to the superior oxidative effects of free radicals not only on nucleic acids but also in other biomolecules [124], even if there are still crucial gaps to be filled regarding the potential spread of AMR [66], and in antibiotic fermentation residues [123].

#### 3.2.2. Non-Thermal or Cold Atmospheric Plasma

Non-thermal or cold atmospheric-pressure plasma is a non-thermal technology used by the food industry as a decontamination technique, with applications varying from the surface or food decontamination and toxin removal to enzyme inactivation, food packaging modifications, and wastewater treatment. Plasma technology belongs to AOP and is based on the ionization of a carrier gas through the application of electric discharges at atmospheric pressure and room temperature, with the consequent generation of electrons, ions, UV photons, charged particles, and free radicals (including reactive oxygen and nitrogen species), which cause direct effects that damage microbial cell membranes, DNA, and proteins [115]. Plasma can inactivate pathogenic microbes, but its ability to induce cross-resistance or co-resistance to clinically relevant antibiotics has yet to be understood. Guo et al. (2018) found that the sublethal treatment of gas plasma decreased the MICs of MRSA against tetracycline, gentamicin, clindamycin, chloramphenicol, ciprofloxacin, rifampicin, and vancomycin and increased persisters eradication, along with increases in the levels of reactive oxygen species and reactive nitrogen species in MRSA cells [125]. Álvarez-Molina and Colleagues (2020) observed a process of adaptation of *E. coli*, *Salmonella* spp. and *L. monocytogenes* strains repeatedly exposed to sublethal non-thermal atmospheric plasma, accompanied by a gradual decrease in bacterial susceptibility in the investigated strains. In addition, a gradual decrease in bacterial susceptibility was reported for *L. monocytogenes* (versus ciprofloxacin and streptomycin), *S*. Typhimurium (versus ciprofloxacin), and *E. coli* (versus tetracycline) variants. Among the *S*. Typhimurium variants obtained through exposure to plasma, one suffered a transition from a ciprofloxacin-susceptible to -resistant phenotype. These variants may be generated through adaptive mutagenesis and, even if the mutagenic potential of plasma is not known, the UV photons and oxidizing free radicals might cause point mutations and rearrangements in the DNA sequence, with the only exception being the resistance profile obtained for the variant strain *S*. Typhimurium versus streptomycin, which is at least partly associated with multidrug efflux. In addition, the mapping of the genome of *S*. Typhimurium and the variant versus ciprofloxacin and streptomycin evidenced a common evolution for various generations, followed by a parallel divergent evolution, which gave rise to relevant phenotype shifts, with different genes harboring non-synonymous mutations in the resistant variants [115].

## 4. Influence of Bacteriophage Application on Antibiotic Susceptibility

Bacteriophage (phage) are the most abundant types of life on earth. They are completely dependent on their host for activity; therefore, they could be one of the most relevant biological stressors for bacteria, namely for foodborne bacteria [126,127,128].

In the widespread bacterial resistance era, the reuse of phage has prospered. They are known to treat infections in humans and animals or even to be used in packaged foods, as an alternative to antimicrobial components [129]. The application of phage has been supported by its advantages, such as its presenting fewer adverse effects on commensal microbiota, being harmless to farm animals and humans, and its interesting effectiveness against antibiotic-resistant bacteria. The proper application of phage in the food chain, from farm to fork, can be considered as a replacement or complement of the use of antibiotics and pesticides. However, based on expert opinions, the route of administration, dosage, and type of phages are very important for the outcome of phage therapy. Nonetheless, this approach needs to be further and more deeply investigated [126,130].

Phage may act on resistant bacteria or work synergistically with antibiotics; moreover, phage selection may also result in bacteria developing phage-resistance, simultaneously increasing susceptibility to antibiotics (trade-off) or leading to modifications associated with cross-resistance to antibiotics [126,131,132].

In this regard, different results have been presented in various works. In some studies, phage presented a trade-up on bacteria, and their use led to resistance against the same and other phages strains, as well as to antibiotics, due to the cross-resistance phenomenon. Reversely, the application of some antibiotics could also induce bacteria to resistance against phage. In contrast, other authors observed the trade-off between phage species and antibiotics and proposed an increased sensitivity to antibiotics under the influence of phage [126,132,133]. Allen et al. (2017) explored the association among antibiotic and phage resistance phenotypes in natural and clinical populations. The authors showed that emerging resistance against one factor did not affect the emergence of resistance in another, with susceptibility to phages and antibiotics evolving independently [134]. In general, resistance to bacteriophage may potentially lead to resistance or sensitivity to a class of antibiotics; however, it is not yet clear which direction is more prevalent in bacteria.

Considering this, bacteriophage may have a differential effect on antibiotic resistance; firstly, antibiotic resistance may be induced due to phage selection pressure. When a bacterium encounters a phage, it tries to survive by several known mechanisms, with some of them being able to influence the efficacy of antibiotics and confer resistance to them. This may occur through an increase in the expression of some genes which are involved in the enzymatic activity or an increase in the thickness of the extracellular layer, modification of LPS, receptors, or outer membrane structures, and blocking or loss of some transport proteins channels (such as porin) that may act simultaneously as phage and antibiotic receptors [126,135,136]. Another important mechanism, which is involved in the antibiotic resistance induced by phage, is the transfer of ARGs between bacteria via transduction [134,137]. Phage can harbor some ARGs, such as *bla_TEM_*, *bla_CTX-M-1_*, *bla_CTX-M-9_*, *bla_OXA-48_*, *bla_VIM_*, *qnrA*, *qnrS*, *mecA*, *armA*, and *sul1* genes, and distribute them between different bacteria [137]. As a cross-resistance example, Moulton-Brown et al. (2018) showed that, when *P. aeruginosa* becomes resistant to phage 14/1, it simultaneously increases survival to the exposure to gentamycin [136]. Another study showed that *Klebsiella* phages can lead to the overexpression of some genes in this bacterium, including *aac(6′)-Ib-cr*, which is responsible for aminoglycoside and quinolone resistance, and the *vagC* gene, which increases β-lactamase activity. PBKP35 phage was able to induce cross-resistance in *Klebsiella pneumoniae* (*K. pneumoniae*) isolates by decreasing the sensitivity level to several classes of antibiotics. A percentage of halo reduction was observed for disk diffusion assay with cefotaxime (78%), cephalothin (76%), kanamycin (74%), chloramphenicol (70%), ciprofloxacin (38%), levofloxacin (38%), erythromycin (21%), and nalidixic acid (14%) [135]. Wang et al. (2010), also suggested a similar result, showing that superinfecting bacteriophages may be associated with resistance to quinolone and β-lactam antibiotics in bacteria, via proteins involved in the cell division inhibition [138]. Another investigation suggested that resistance against T6 phage infection in *E. coli* can induce cross-resistance against gentamicin, as *tsx* gene deletion, a gene that encodes the nucleotide channel used as a receptor by the phage, confers resistance to the phage and simultaneously increases resistance to gentamicin [134].

The modification of LPS, possibly due to the stress pressures of phages infection or β-lactams exposure, changes the structure of porin (OmpF)-LPS complexes. These modifications involve an alteration of the cell membrane proteins, including β-lactams and phage receptors, on the outer membrane of Gram-negative bacteria. Alterations in those structures contributes to resistance against phage or antibiotics. Therefore, resistance to one of them may fortuitously confer cross-resistance to the other one [126]. For example, infection of *E. coli* with T3 phage leads to the emergence of resistant mutants with LPS modifications that are also resistant to amoxicillin. Conversely, T3 phage-resistant mutants can be developed following exposure to a sublethal dose of amoxicillin [126]. Theoretically, Gram-positive MDR bacteria can emerge with the same mechanism via the modification of teichoic acids, as a known phage receptor [139].

In contrast to these studies, other researchers proposed the reduction in antibiotic resistance by phage interactions. One approach to this is the influence of phages in the restoration of antibiotic sensitivity by modifying the bacterial drug efflux pumps. In fact, phages use extracellular peptide loops of TolC or OprM, the outer membrane subunits of RND family efflux pumps, as binding sites, to enter and infect their hosts. Bacterial mutants try to reduce the number or modify the structure of efflux pumps to prevent phage’s entering, which may pleiotropically reduce the efficacy of these multidrug efflux systems against antibiotics and, as a result, increase the bacterial antibiotic susceptibility [131,132,139,140,141]. Chan et al. (2016) proved that one of the receptors of a *P. aeruginosa* phage is OprM, the outer membrane protein from the multidrug efflux systems MexAB and MexXY, and this protein has suffered changes in some mutants (phage resistance), which could decrease the resistance level of several antibiotic classes [132]. Gurney et al. (2020) also showed that the long-term exposure to phage OMKO1 can decrease the MIC level of antibiotic resistance in more than 60% for *P. aeruginosa* (Washington PAO1) against tetracycline, erythromycin, and ciprofloxacin by the reduction in efflux pump efficiency [142]. Supporting this, *E. coli* TLS phage-resistant mutants suffer a change in the AcrAB-TolC system that might pleiotropically lead to a loss of antibiotic resistance [131,140]. U136B phage also uses TolC as a receptor to infect *E. coli*, which was related to a trade-off between phage resistance and antibiotic resistance, with *tolC* mutants associated with phage-resistance being more sensitive to tetracycline and colistin in comparison to their parental strains. Meanwhile, a subset of the other phage-resistant mutants with LPS-related mutations showed decreased resistance to colistin, while presenting increased resistance to tetracycline, which indicates the variability in phage-antibiotic resistance interaction in bacteria [131,142]. Hao et al. (2019) found a similar trade-off in the complementary direction, with strains with increased colistin resistance showing a reduced phage resistance, which indicates that this trade-off can evolve in both directions [143]. In contrast, LPS-related changes in phage-resistant mutants may reduce the expression of OmpF, tetracycline’ uptake porin, and lead to negative consequences in pathogenic bacterial populations by increasing tetracycline resistance [131,144].

In sum, increasing knowledge of the interaction between bacteria and phage is necessary to evaluate phage application in the food chain and reduce the cross-resistance phenomena between antibiotics and phages. By accurately identifying candidate phages in each foodborne bacterium, with both trade-off and synergistic effects with a class of antibiotics, a therapeutic combination of both types of antibacterial agent can be developed against MDR bacteria. However, despite the progress in the understanding of associations between antibiotics and phage in natural environments, animal farms, and clinical populations, we still have little information in this regard. Therefore, in any phage therapy protocol and study, particular attention should be paid to cross-resistance, unexpected pleiotropy, and community evolution.

## 5. Conclusions

Antimicrobial resistance in the food chain can be influenced by several factors, such as the horizontal ARGs transfer or the induction of AMR related to responses directed to diverse food chain stresses, namely by sublethal exposure or recurring exposure to: (i) an extensive diversity of antimicrobial agents, for example, the disinfectants used in several steps of the food chain, but also agrochemicals or even food preservatives; (ii) different physical treatments used in food processing, such as thermal and non-thermal treatments; (iii) new food safety approaches, such as the use of bacteriophage as an alternative to antibiotics in animal health or as biopreservatives. These co-selection or cross-adaptation phenomena are in need of further study, due to their complexity and relevance as a public health concern. Despite various works addressing this topic, a very limited group of foodborne pathogens has been covered, and different conclusions were obtained, meaning that some points are still inconclusive. In addition, the lack of critical evaluations of the specific mechanisms involved in antimicrobial resistance and physical-chemical treatments hinders the estimation of the real risk of antibiotic resistance. This is a major drawback in the balance between the desired use of physical–chemical treatments, chosen by the food industry to assure food safety, and the evidence that these same treatments do guarantee safety in terms of bacterial antibiotic resistance. Furthermore, a thorough understanding of the limitations in the bacterial population involved in these studies, in terms of the variability of strains among each considered bacterial species, the different food and environmental sources, and the heterologous patterns of antibiotic susceptibility/resistance, is essential to guide future research. Thus, it is of paramount importance to address cross-resistance or co-resistance events as a food safety issue, along with studying these effects during the experimental stage of evaluation of novel control strategies, but also of the recurrently used in the food chain.

## Figures and Tables

**Table 1:** Examples of bacterial cross-adaptation to antibiotics induced by exposure to non-antibiotic antimicrobials.

Non-Antibiotic Antimicrobials	Bacterial Species	Antibiotics to which Susceptibility was Decreased	Bacterial Adaptation/Mechanism of Resistance	Reference
Agrochemicals				
Combination of three pesticides: captan, carbaryl, and malathion	*S. aureus*	Sulfamethazine	Not reported	[11]
Dicamba	*E. coli*/ *S.* Typhimurium	Chloramphenicol, ciprofloxacin, tetracycline/ Ampicillin, chloramphenicol, ciprofloxacin, tetracycline		[12]
2,4-dichlorophenoxyacetic acid		Ampicillin, ciprofloxacin/ Ampicillin, chloramphenicol, ciprofloxacin, tetracycline	Efflux pumps and induction of the *soxRS* regulon account for the change in susceptibility in *E. coli*. Dicamba plus chloramphenicol and Roundup plus kanamycin	
Glyphosate (RoudUp)		Ciprofloxacin/ Ciprofloxacin, kanamycin		
Mixture of 23 pesticides	*E. coli*	Streptomycin	Mutations associated with the antibiotic target	[13]
**Biocides**				
Sodium hypochlorite	*E. coli*	Spectinomycin, nalidixic acid, ampicillin-sulbactam	Increase in cell surface hydrophobicity and biofilm formation, changes in cell morphology and ultrastructure	[14]
Quaternary ammonium disinfectant or triclosan	*S*. Typhimurium	Chloramphenicol, ciprofloxacin, tetracycline, ampicillin	Overexpression of AcrAB efflux pump and reduction in outer membrane porins	[15]
Triclosan	*E. coli*	Ampicillin, ampicillin-sulbactam, cefazoline, cefaclor, cefotaxime, cefepime, erythromycin, azithromycin, gentamicin, chloramphenicol, tetracycline, ciprofloxacin, lomefloxacin, imipenem	Changes in bacterial membrane properties and enhancing the efflux system	[16]
Quaternary ammonium disinfectant	*S. aureus*	Fluoroquinolones	Increased expression of *norA*	[17]
Quaternary ammonium disinfectant (benzalkonium chloride)	*L. monocytogenes*	Cefotaxime, cephalothin, ciprofloxacin	Increased expression of MdrL efflux pump	[18]
Benzalkonium chloride or chlorhexidine	*P. aeruginosa*	Ciprofloxacin, novobiocin	Decrease in the expression of the repressor gene *mexR* and increase the activity of MexAB-OprM and MexCD-OprJ efflux pumps	[19,20]
Didecyldimonium chloride	*P. aeruginosa*	Colistin, ceftazidime, amikacin, meropenem, gentamicin, piperacillin-tazobactam, ciprofloxacin	Not reported	[21]
Sodium hypochlorite	*P. aeruginosa*	Amikacin, gentamicin, meropenem, ciprofloxacin	Not reported	[21]
Chlorhexidine	*K. pneumoniae*	Colistin	Mutations in Tet repressor gene (*smvR*) and up-regulation of the *smvA* gene, both involved in MFS efflux pump system; modification of LPS	[22]
Chlorine	*Salmonella* Enteritidis	Tetracycline, nalidixic acid, chloramphenicol	MarRAB operon and increased expression of efflux pumps	[23]
Chlorine	*S. enterica* serovar Heidelberg	Gentamicin, streptomycin, ampicillin, ciprofloxacin (adapted rugose); sulphamethoxazole/trimethoprim and streptomycin (adapted smoothly)	Not reported	[24]
Chlorine	*E. coli*	Trimethoprim	Not reported	[25]
Chlorine (>1.0 mg Cl2/L)	*E. coli*	Tetracycline	Not reported	[26]
Chlorine (2 mg/L)	*E. coli*	Ampicillin	Not reported	[27]
Chlorine (1 and 5 mg/L)	*K. pneumoniae*	Ampicillin	Not reported	[28]
Chlorine (4 and 8 mg/L)	*P. aeruginosa*	Ceftazidime, chloramphenicol, ampicillin	Not reported	[29]
Chlorine	*P. aeruginosa*	Amikacin, gentamicin	Not reported	[21]
**Heavy Metals**				
Cr Pb Cd Zn Cu	*E. coli*	Fluoroquinolone Vancomycin Quinolone Fluoroquinolone, ampicillin, cephalothin, and trimethoprim/sulfamethoxazole, vancomycin Ampicillin, cephalothin, trimethoprim/sulfamethoxazole	Not reported	[30]
Hg	Enterobacteriaceae	Various antibiotics (not specified)	Not reported	
Co, Cr, Cu, Hg, Ni, Zn Pb	*Salmonella* spp.	Penicillin Ampicillin, chloramphenicol, tetracycline		
Ag, Cd, Cu, Ni, Pb, Zn Zn, Cu	*P. aeruginosa*	Aminoglycoside, amphenicol, macrolide, nitrofuran, penicillin, Quinolone, sulfonamide, tetracycline, trimethoprim/sulfamethoxazole; imipenem	Outer membrane proteins Co-regulation	
Cd	*A. baumannii, Klebsiella* spp., *P. aeruginosa, Providencia* spp, *Proteus* spp.	Penicillin, ampicillin	Not reported	
Hg	*E. coli, Klebsiella* spp., *Shigella* spp.	Tetracycline, Sulfamethoxazole/trimethoprim	Not reported	
Cu, Ni, Zn	*Klebsiella* spp., *P. aeruginosa, Proteus* spp.	Ampicillin, amoxicillin, tetracycline	Not reported	
**Food preservatives and decontaminants**				
Lactic acid	*Cronobacter sakazakii*	Neomycin, tetracycline, tilmicosin, florfenicol, Amoxicillin, ampicillin, vancomycin, ciprofloxacin, enrofloxacin	Not reported	[31]
Acidification with HCl	*E. coli* *S.* Typhimurium *S. aureus*	Amikacin, ceftriaxone, nalidixic acid Amikacin, ceftriaxone, trimethoprim Gentamicin, erythromycin	Not reported	[32]
Acetic acid, sodium benzoate, sodium nitrite	*S.* Enteritidis	Tetracycline	*mar* mutation	[23]
Trisodium phosphate	*E. coli*	Ampicillin	Not reported	[14]
Sodium nitrite		Spectinomycin, amikacin, kanamycin, streptomycin, cefazolin, cephalothin, cefotaxime, ceftazidime, cefepime, aztreonam, nalidixic acid, enrofloxacin, phosphomycin, nitrofurantoin	Increase in cell surface hydrophobicity and biofilm formation	
Lactic acid (pH 6, 5.5, 5)	*L. monocytogenes*	Streptomycin, gentamicin, ampicillin, penicillin, ciprofloxacin, enrofloxacin	Not reported	[33]
Lactic acid (1%, pH 3.5)	*L. monocytogenes*	Ciprofloxacin, nitrofurantoin, erythromycin	Not reported	[34]
Sulphuric acid (pH 3, 5, 6)	*Acinetobacter baumannii*	Amikacin, piperacillin, tazobactam, imipenem, meropenem	Not reported	[35]
**Natural Compounds**				
Epigallocathechin gallate	*S. epidermis, S. aureus*	Vancomycin, oxacillin, ampicillin	Increased cell wall thickness, with a role of the two-component VraSR system	[36,37]
*Melaleuca alternifolia* oil	*E. coli*	Gentamicin, erythromycin, vancomycin, chloramphenicol, tetracycline, trimethoprim, mupirocin	Not reported	[38]
	*S.* Enteritidis, *S.* Typhimurium	Gentamicin, chloramphenicol, tetracycline, streptomycin, trimethoprim, mupirocin		
	*S. aureus*	Gentamicin, vancomycin, chloramphenicol, trimethoprim, ampicillin, fusidic acid, mupirocin		
*Thymus marroccanus* essential oil	*E. coli*	Chloramphenicol, nalidixic acid, tetracycline, erythromycin	Overexpression of AcrAB-tolC and decrease of the expression of outer membrane proteins	[39]
Pine oil	*E. coli*	Tetracycline, ampicillin, chloramphenicol	Overexpression of marA gene.	[40]

## Data Availability

Data is contained within the article.
